# TNF-α enhances sensory DRG neuron excitability through modulation of P2X3 receptors in an acute colitis model

**DOI:** 10.3389/fimmu.2022.872760

**Published:** 2022-08-12

**Authors:** Eduardo E. Valdez-Morales, Carlos A. Sánchez-Navarro, Diana Reyes-Pavón, Tonatiuh Barrios-Garcia, Fernando Ochoa-Cortes, Alma Barajas-Espinosa, Paulino Barragán-Iglesias, Raquel Guerrero-Alba

**Affiliations:** ^1^ Facultad de Medicina y Cirugía, Universidad Autónoma “Benito Juárez” de Oaxaca, Oaxaca, Mexico; ^2^ Departamento de Medicina, Centro de Ciencias de la Salud , Universidad Autónoma de Aguascalientes, Aguascalientes, Mexico; ^3^ Departamento de Fisiología y Farmacología, Centro de Ciencias Básicas, Universidad Autónoma de Aguascalientes, Aguascalientes, Mexico; ^4^ Licenciatura en Enfermería, Escuela Superior de Huejutla, Universidad Autónoma del Estado de Hidalgo, Hidalgo, Mexico

**Keywords:** Colitis, DRG neurons, hyperexcitability, P2X3 receptor, TNF-α

## Abstract

Previous studies have demonstrated that acute colonic inflammation leads to an increase in dorsal root ganglia (DRG) neuronal excitability. However, the signaling elements implicated in this hyperexcitability have yet to be fully unraveled. Extracellular adenosine 5’-triphosphate (ATP) is a well-recognized sensory signaling molecule that enhances the nociceptive response after inflammation through activation of P2X3 receptors, which are expressed mainly by peripheral sensory neurons. The aim of this study is to continue investigating how P2X3 affects neuronal hypersensitivity in an acute colitis animal model. To achieve this, DNBS (Dinitrobenzene sulfonic acid; 200 mg/kg) was intrarectally administered to C57BL/6 mice, and inflammation severity was assessed according to the following parameters: weight loss, macroscopic and microscopic scores. Perforated patch clamp technique was used to evaluate neuronal excitability *via* measuring changes in rheobase and action potential firing in T8-L1 DRG neurons. A-317491, a well-established potent and selective P2X3 receptor antagonist, served to dissect their contribution to recorded responses. Protein expression of P2X3 receptors in DRG was evaluated by western blotting and immunofluorescence. Four days post-DNBS administration, colons were processed for histological analyses of ulceration, crypt morphology, goblet cell density, and immune cell infiltration. DRG neurons from DNBS-treated mice were significantly more excitable compared with controls; these changes correlated with increased P2X3 receptor expression. Furthermore, TNF-α mRNA expression was also significantly higher in inflamed colons compared to controls. Incubation of control DRG neurons with TNF-α resulted in similar cell hyperexcitability as measured in DNBS-derived neurons. The selective P2X3 receptor antagonist, A-317491, blocked the TNF-α-induced effect. These results support the hypothesis that TNF-α enhances colon-innervating DRG neuron excitability *via* modulation of P2X3 receptor activity.

## Introduction

Both Crohn’s disease (CD) and ulcerative colitis (UC) are types of inflammatory bowel disease (IBD) that affect approximately 1 million young (15-35-year-old) people in the USA and 2.5 million in Europe (1). Both are chronic diseases characterized by recurrent GI-tract inflammation, yet they differ in their GI area distribution and inflammation severity. Whereas CD affects the entire GI tract indiscriminately (although it is most common in the terminal ileum or the perianal region) and causes damage to the whole thickness of the affected tissue, UC primarily affects the mucosal and submucosal layers and is limited to the colon and presents both flare-ups (active phase) and periods of remission (inactive phase) (2). Dysregulated immune responses in the intestinal mucosa are critical factors in the onset of IBD (3). A steady release of pro-inflammatory mediators, including TNF-α, during flares leads to sensitization of gut-innervating nociceptive neurons in the dorsal root ganglia (DRG), resulting in visceral pain (4). This increase in nociception has been observed during acute flare-ups in UC patients (5).

There is an increasing body of evidence that extracellular adenosine 5’-triphosphate (ATP) is an important inflammatory mediator capable of directly exciting nociceptors (6). ATP elicits this effect by activating ligand-gated cation channels denominated P2X receptors, localized at the periphery of sensory neurons and centrally on the second-order neurons of the dorsal horn (7). Seven P2X receptors subunits (P2X1-P2X7) have been identified. The P2X3 receptor subtype is primarily expressed in small and mid-sized DRG neurons, classified as nociceptive C-fiber primary afferent neurons (8). Upon ATP binding to the P2X3 receptors, Na^+^ and Ca^2+^ ions are mobilized, inducing membrane depolarization, leading to activation of Ca^2+^-dependent intracellular processes, conducing to pain and hyperalgesia (7).

The role of P2X3 receptors in pain and hypersensitivity have been extensively studied, and they are widely accepted as mediators of abnormal pain responses post-spinal cord injury or inflammation (9). Activation of homomeric P2X3 or heteromeric P2X2/3 receptors by endogenous ATP has been demonstrated to be pivotal for the development of hyperalgesia under inflammatory conditions (10). It is perhaps, thus, not surprising that the expression of these receptors is upregulated in IBD patients’ colon biopsies (11). The P2X3 receptor has also been implicated as a mediator of visceral hypersensitivity during acute chemically-induced colitis (12). However, the molecular mechanisms by which the P2X3 receptor mediates this visceral hypersensitivity are not well understood. Therefore, the present study aimed to determine whether pro-inflammatory mediators released during acute experimental colitis directly modulate P2X3 receptors inducing hyperexcitability of DRG neurons.

## Materials and methods

### Animals

Male, 6-10 weeks old C57BL/6 mice obtained from the Laboratory Animal Service of the Autonomous University of Aguascalientes were used in this study. Animal protocols followed the guidelines from the Mexican norm (NOM-062-Z00-1999) and were approved by the Ethics Committee Concerning Animals in Teaching and Research at the Autonomous University of Aguascalientes (CEADI-UAA 5/03/2018). Mice were randomly divided into two groups of five and maintained at an ambient temperature of 23-25°C, with 12 light/12 dark cycles, and fed *ad libitum* (Nutricubo, Purina USA).

### Acute colitis animal model

Prior to colitis induction, mice were fasted overnight (16-18 hours). Animals were anesthetized by isoflurane inhalation, and colitis was induced *via* intrarectal administration of DNBS (100 μl, 2-6 mg; dissolved in 50% ethanol (EtOH; v/v)), deposited with a polyethylene catheter, 3 cm into the colon, as previously described (13). The control group received only 50% EtOH (v/v) intrarectal administration. Four days post-DNBS application, the mice were euthanized with a sodium pentobarbital overdose (200 mg/kg).

### Colon inflammation assessment

In order to discern the inflammation severity caused by DNBS, the following parameters were recorded daily: body weight, stool consistency, and fecal blood. The difference between the daily recorded weights and the initial body weight (prior to DNBS administration, taken as the 100% weight) was determined to be the % weight loss induced by colitis. As previously described, stool consistency was determined (see **Table 1**), and fecal blood was detected using Hema screen test strips (14).

**Table 1:** The score of diarrhea and visible fecal blood.

Score	Diarrhea score	Fecal blood
0	Normal pellets	None present
1	Slightly loose feces	Slightly bloody (occult blood)
2	Loose feces	Adherence of blood around the anus
3	Watery diarrhea	Bleeding through the anus

Upon completion of the study, mice were euthanized, the distal colon was exteriorized, dissected free, rinsed with cold PBS, and photographed for posterior serosal-surface damage analyses according to the validated criteria described by Morris and collaborators (15), where 0 = no bleeding, no ulcers or edema; 1 = mild bleeding, mild edema, or mild mucosal erosion; 2 = moderate edema, ulcers or bleeding erosions; 3 = severe ulceration, erosions, edema, tissue necrosis, and perforation. The damage analyses (scoring) were conducted by two independent individuals blinded to the treatment of the mice. The dissected colon was divided into three 1 cm segments and used as follows.

The first piece of colon was fixed in 10% formalin for 24 hours (Sigma-Aldrich, Inc. St. Louis, USA), dehydrated in graded alcohols, cleared in xylene, and embedded in paraffin wax. Colon cross-sections (7 μm thick) stained with hematoxylin and eosin (H & E) were visualized under a light microscope (Axioscope 40, Carl Zeiss, Germany) and photographed for further analysis.

A second colon section was employed for the myeloperoxidase (MPO) assay, a well-established assay for determining neutrophil presence and activity (16). This assay is commonly utilized to determine the level of inflammation in colon tissue in different colitis models (17, 18). To perform this assay, a 1 cm distal colon section was weighed and homogenized (D1000 homogenizer, Benchmark Scientific MA, USA) in 0.5% hexadecyltrimethylammonium bromide (HTAB) buffer made in potassium phosphate (50 mM, pH 6.0). Homogenates were centrifuged at 13,400 g, 10 min at 4°C, and supernatants were aliquoted and stored. A total volume of 100 μl of each supernatant was added to 2.9 mL of O-dianisidine solution (16.7 mg/mL of O-dianisidine dihydrochloride and 0.5% hydrogen peroxide in PBS). Absorbances at wavelength of 450 nm were measured using a microplate reader (iMark, Biorad, Hercules CA, USA) during a time-lapse of 60 s. Data were expressed as units per gram of colon (U/mg), where one enzymatic unit is defined as the amount of myeloperoxidase that can hydrolyze 1 μmol hydrogen peroxide in 60 s at room temperature.

Finally, from the third colon segment, RNA was isolated, and expression levels were determined, by semi-quantitative RT-PCR, for the following transcripts: tumor necrosis factor-alpha (TNF-α), interleukin-1beta (IL-1β), and interleukin-6 (IL-6).

### Analysis of pro-inflammatory cytokines gene expression

Using TRIzol-Chloroform (Invitrogen, Carlsbad, CA, USA), RNA was extracted from dissected 1 cm colon sections. Total RNA obtained from all colon samples was quantified with a Nanodrop 2000 spectrophotometer (Thermo Scientific, U.S.A), and RNA quality was calculated based on the absorbance ratios at 260/280 and 260/230 nm; the accepted ratio range was 1.9-2.0 (19). The NanoDrop software automatically calculated the nucleic acid concentration, of which 1 μg total RNA was reversed transcribed with the iScript kit (Biorad, Hercules CA, USA) according to the manufacturer’s instructions. From this, we amplified 1 μl of cDNA/25 μl reaction volume (containing: 1X PCR buffer, 1.5 mM MgCl2, 0.2 mM dNTPs, 0.2 mM of forward and reverse primer, and 0.5 U DNA Taq Polymerase (Life Technologies, TX, USA)). Transcript glyceraldehyde 3-phosphate dehydrogenase (GAPDH) was used as the internal control, and the expression of other transcripts was normalized to it. Each transcript amplification was conducted in triplicate, and the results shown are the average of these three amplifications. Amplification conditions were as follows: 3 min denaturation at 94°C, 35 amplification cycles (denaturation: 30 s at 95°C; annealing: 30 s at 55°C; and extension: 30 s at 72°C). Following the completion of the final cycle, the samples were incubated for an additional 5 min period at 72°C for terminal elongation. All primers were purchased from Integrated DNA Technologies, their sequences are listed in **Table 2**.

**Table 2:** Primer sequences used in RT-PCR assays in colonic tissue and their expected product lengths in base pairs (bp).

Gene	Sequence (5’-3’)	Annealing T(°C)		bp
GAPDH	F - CCA TCA CCA TCT TCC AGG AG R - CCT GCT TCA CCA CCT TCT TG		55	576
TNF-α	F - AAC TAG TGG TGC CAG CCG AT R - CTT CAC AGA GCA ATG ACT CC		50	334
IL-1β	F - TGA TGA GAA TGA CCT CTT CT R – CTT CTT CAA AGA TGA AGG AAA		55	251
IL-6	F - TAG TCC TTC CTA CCC CAA TTT CC R - TTG GTC CTT AGC ACT CCT TC		55	76

A total of 5 μl of each PCR product were separated on a 1.5% agarose gel, stained with 0.1% ethidium bromide, photo-documented (miniBis Pro, DNR Bio-Imaging System), and the densitometry of the visible band determined using Image J 1.43 (NIH) software, following the user´s manual (20). As aforementioned, all products were normalized with the housekeeping control, GAPDH.

### DRG dissection and culture

For DRG isolation, control and DNBS mice were anesthetized with isofluorane inhalation. Subsequently, intracardiac perfusion with Hank’s balanced salt solution (HBSS) allowed the clearing of systemic blood. Following this, laminectomy was performed to dissect the DRGs from levels thoracic 8 to lumbar 1 (T8-L1), known to contain colon projecting nociceptive DRG neurons (21, 22). DRGs were collected in cold HBSS for primary culture. The ganglia were dissociated by enzymatic treatment using a mixture of Trypsin (1 mg/mL) and collagenase D (2.8 mg/mL) and incubating for 30 minutes at 37°C. After this incubation period, enzymes were removed from the tissue by washing with HBSS and 1 ml of F12 medium (Sigma Aldrich^®^) with 100 U/ml penicillin and 0.1 mg/ml streptomycin supplemented with 10% fetal bovine serum was added. Disaggregated neurons were plated on round coverslips previously covered with poly-D-lysine (20 μg/ml) in 24-well dishes in a humid, 37°C incubator with 95% O2 and 5% CO2 atmosphere for 18-24 h before further manipulation. Cultured DRG neurons from naïve mice were incubated with human TNF-α solution (0.1 μg/mL), TNF-α/ATP (100 µM) or F-12 medium alone for 17-18 hours before electrophysiological studies (23).

### Patch clamp recordings

The perforate-patch current clamp recordings with 0.24 mg/ml amphotericin B (Sigma-Aldrich) were performed with borosilicate pipettes with resistance between 2-5 MΩ on small neurons (≤ 40 pF capacitance), which are known to exhibit nociceptive properties (24). DRG neurons were superfused with an external solution containing (mM): 140 NaCl, 5 KCl, 1 MgCl_2_, 2 CaCl_2_, 10 HEPES, and 10 D-glucose, pH adjusted to 7.4 with NaOH. The composition of the internal pipette solution was (in mM): 110 K-gluconate, 30 mM KCl, 10 HEPES, 1 MgCl_2_, and 2 CaCl_2_ with pH adjusted to 7.25 using KOH.

### Immunofluorescence

After euthanizing the animals, the DRGs (T8-L1) from control and DNBS mice were dissected and immediately embedded in O.C.T. on dry ice. Cryosections (20 μm) were adhered on SuperFrost Plus slides (Thermo Fisher Scientific), fixed for 1 hour in ice-cold 10% formalin in 1 X PBS, washed 3x 10 minutes in PBS, and permeabilized for 30 minutes in 0.2% Triton X-100 (Sigma-Aldrich). Following this, excess Triton was washed off 3x 5 minutes with PBS, and tissues were blocked for 2 hours in 10% NGS (normal goat serum, ThermoFisher). Incubation with Alexa Fluor 568-labeled isolectin IB4 (cat # I21412; Thermo Fisher) and P2X3 antibody (1:500; cat # ab10269; Abcam) was performed overnight at a temperature of 4°C. Finally, a 1-hour incubation with Alexa Fluor 488- conjugated secondary antibody was performed at room temperature (approximately 21°C). A P2X3-positive nociceptive neuron subgroup found in the DRG has been previously identified to also bind this isolectin; thus, both markers were hereby employed with the purpose of identifying these cells (24–26). Fluorescent emission was visualized and images were captured using an Olympus FluoView 1200 confocal microscope; colocalization of P2X3 and IB4 analysis was performed as described previously (27). Colon samples of 3 separate animals per treatment group (control and DNBS) were processed as described, and representative images (projections of z stacks) were presented in the results. Using FIJI (Image J), the corrected total cell fluorescence (CTCF) was calculated to determine the intensity of the signal between experimental groups. To do so, the integrated density and the area, as well as the background noise, were measured, and the CTCF was calculated as equal to the integrated density - (area of selected cell x mean fluorescence of background readings). CTCF values from the DNBS-treated group were normalized to the control group and expressed as normalized CTCF.

### Western blotting analysis

Western blotting was used to examine the expression of P2X3 receptor and GAPDH expression in DRG (T8-L1) neuron protein extract. A total of six mice per group (control vs DNBS) were used for DRG harvesting. Total ganglia (T8-L1) from each mouse were pooled together, snapped frozen on dry ice, and later homogenized in 300 μl of lysis buffer (50 mM Tris, pH 7.4; 150 mM NaCl; 1 mM EDTA, pH 8.0, and 1% Triton X-100) supplemented with protease (P8340 Sigma-Aldrich^®^) and phosphatase (P5726 and D0044 Sigma-Aldrich^®^) inhibitors. Homogenates were centrifuged at 14,000 rpm, 15 min at 4°C; the supernatant was isolated and stored at -80°C. Protein quantification was conducted with the Micro BCA protein assays kit (Thermo Scientific, Rockford, IL). A total of 15 μg of protein was separated in a 10% SDS-PAGE and transferred onto PVDF membranes (Biorad, Hercules CA, USA) overnight at 25 V and at 4°C. Non-specific antibody binding was blocked by incubating the membrane for at least 24 hours with 5% nonfat dry milk in 1X Tris Buffer solution containing Tween 20 (TTBS). Excess milk was washed off with 1X TTBS, and membranes were then incubated with anti-P2X3 (1:1000; Abcam #ab10269) and GAPDH (1:10,000; Cell Signaling) overnight at 4°C. The following day, the primary antibody was removed, excess washed off with 1X TTBS, and membranes were then incubated with the corresponding secondary antibody at room temperature for 1 hour (1:10,000; Jackson Immunoresearch). After incubation, membranes were washed with 1X TTBS, and immunoreactivity was detected using Clarity™ Western ECL substrate (BioRad) and documented with MicroChemi 4.2 (Bio-imagine Systems). Densitometric analysis was performed using LI-COR Image Studio Software.

### P2X3 receptor antagonism

DRG cells were incubated with P2X3 receptor antagonist, A-317491, 30 min before the application of ATP or TNF-α, unless otherwise mentioned.

### Solutions and reagents

F12 medium, Fetal Bovine Serum (FBS), L-glutamine, penicillin, streptomycin, ATP, A-317491, human TNF-α, and all salts were purchased from Sigma-Aldrich (Toluca, MX). ATP stock solution (100 mM) was made using deionized distiller water and stored frozen; these were diluted to obtain the desired final concentration in external solution prior to use, and the pH was adjusted to 7.4 with NaOH.

### Statistical analysis

Data values are presented as the mean ± standard error of the mean (S.E.M.). The number of performed experiments is designated as “n.” Cells from at least five different mice were used for each experimental protocol. To test statistical differences between two data sets, we used the unpaired Student’s t-test. One-way ANOVA with Bonferroni’s *post hoc* test was used for multiple comparisons. Data were considered statistically different when P ≤ 0.05 in GraphPad Prism version 8 (GraphPad Software, La Jolla, CA, USA).

## Results

### Acute colitis development

**Figure 1:**
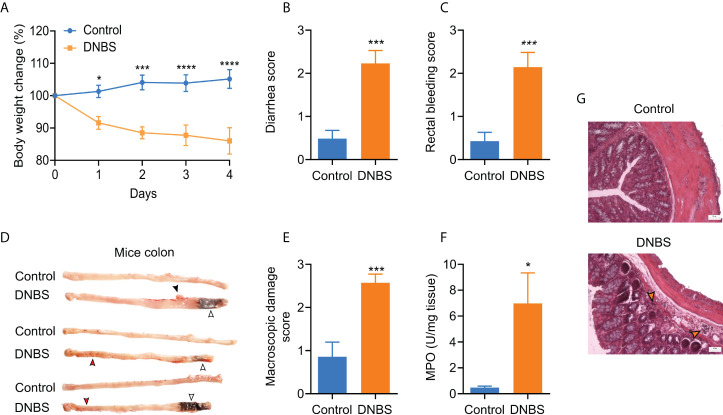
Assessment of acute DNBS colitis. **(A)** Body weight changes posterior to intrarectal DNBS application. Body weight change was calculated as the percent difference relative to initial body weight. **(B)** Diarrhea score. **(C)** Bleeding score. **(D)** Representative colon images of mice in the two groups. **(E)** Macroscopic damage score. **(F)** MPO activity was increased in DNBS-treated mice in comparison with control mice. **(G)** Representative H & E-stained colon sections from control and DNBS mice; the orange arrowhead indicates the cellular infiltrate; 50 µm; magnification x10. Data are presented as the mean ± S.E.M, n =5 -7 per group. DNBS administration was associated with a significant decline in body weight and diarrhea, bleeding, and macroscopic damage score. * P < 0.05, ***P < 0.001 and **** P < 0.001. For body weight change, the statistical analysis by two-way ANOVA was followed by Bonferroni’s post hoc test. For the scores, the statistical analysis was an unpaired t-student test.

The presence of colitis is generally confirmed using body weight loss, diarrhea, rectal bleeding, and macroscopic damage scores. As expected, colitis induction produced significant body weight loss in the DNBS group (**Figure 1A**), reaching 14% after four days. Furthermore, diarrhea and rectal bleeding scores were significantly higher (*P* ≤ 0.001) for the DNBS-treated group than for the control group (**Figures 1B**, **C**). Four days after DNBS application, the mice were euthanized, and their colons were dissected, scored, and processed. Visual inspections of the colon revealed erosions, adhesions, colon wall thickness, and petechial hemorrhage in all animals treated with DNBS (**Figure 1D**), resulting in a macroscopic damage score 2.9 times greater in DNBS-treated mice compared to controls (*P* = 0.001; **Figure 1E**). In addition, histological analysis of the distal colon revealed occasional epithelial erosions and extensive immune cell infiltration in DNBS colons, neither of which were present in the controls (**Figure 1G**). Neutrophil infiltration, assessed indirectly *via* MPO tissue activity content, is commonly accepted as a positive indicator of acute inflammation (17, 18). DNBS administration resulted in colon MPO activity ~17-fold greater, compared to the control group (7.0 ± 2.3 U/mg *vs* 0.4 ± 0.1 U/mg; *P* < 0.05; n = 5; **Figure 1F**). Taken together, these results indicate that DNBS- treated mice exhibited significant signals of acute colitis.

### Colitis-induced increased TNF-α mRNA expression

**Figure 2:**
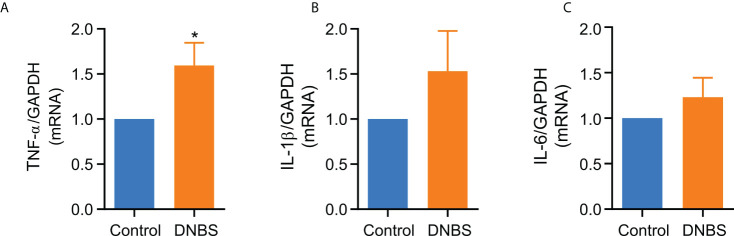
TNF-α mRNA levels are elevated in colonic tissue from DNBS-induced colitis mice compared with control mice. **(A)** TNF-α **(B)** IL-1β and **(C)** IL-6 mRNA levels in colonic tissue from mice of two groups. Data are expressed as mean ± S.E.M (n = 8-9). Statistical differences * P < 0.05 as compared with the control group by unpaired t-student test.

The colonic inflammatory process induced by DNBS in mice was further reflected by a statistically significant, 1.5-fold increase in TNF-α mRNA expression compared to the control group (*P* < 0.05; **Figure 2A**). However, even when there was an increase in IL-1β and IL-6 mRNA expression in colons of DNBS-treated mice compared to controls, this difference was not statistically significant (**Figures 2B, C**). These results indicate that colonic inflammation in this acute model is influenced primarily by TNF-α overexpression.

### Acute colitis leads to hyperexcitability of DRG neurons

**Figure 3:**
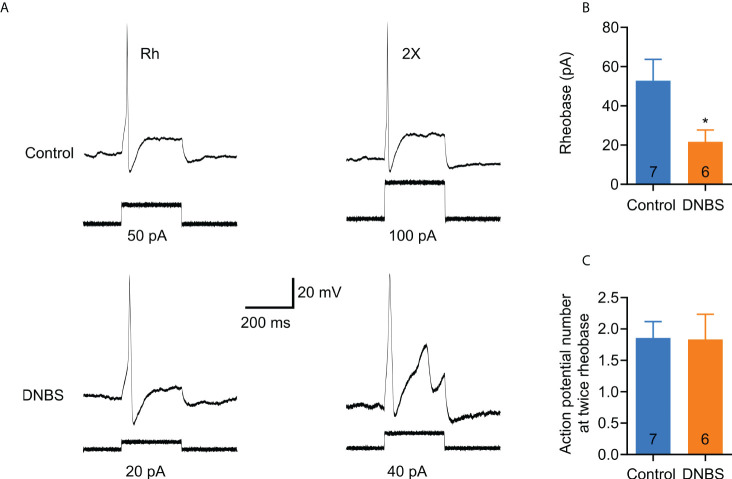
T8-L1 DRG neurons isolated from DNBS-treated mice are more excitable than those isolated from control mice. **(A)** Representative patch clamp recordings of an isolated neuron from a DNBS-treated mouse showed decreased rheobase (lower left trace) and an isolated neuron from a control mouse (upper left trace). The traces of the right (upper and lower) are the action potentials at twice the found rheobases. **(B)** Bar graphs showing the rheobase data. Cells isolated from DNBS mice have a marked decrease in rheobase compared to cells from control mice (*P < 0.05). The number inside each bar on the graph shows the number of cells recorded obtained from at least three different mice. The bars represent the mean ± the S.E.M. The * indicates statistically significant by the unpaired t-student test. **(C)** Bar graph of the summary data of the number of action potentials at twice the rheobase. No significant changes are observed. The numbers inside the bars represent the number of neurons recorded, and the bars represent the mean ± SEM.

To study the effect of acute colitis on neuronal excitability, we cultured T8-L1 DRGs from DNBS-treated and control mice. The current clamp recording trace shown in **Figure 3A** is representative of DRG membrane potential at rheobase (minimum current stimulus required to elicit a single action potential) and twice rheobase current injection. The rheobase was significantly reduced (41%) by DNBS treatment (DNBS: 21.7 ± 6.0 pA *vs* Control: 52.8 ± 10.8 pA; *P* = 0.035; **Figure 3B**). However, mean action potential discharge at twice rheobase was similar between the DNBS and control groups (**Figure 3C**). Neuronal resting membrane potential was alike in both treatment groups (control: -45.6 ± 1.29 mV *vs* DNBS: -46.7 ± 1.22 mV), nor input resistance (control: 880.69 ± 87.18 MΩ *vs* DNBS: 926.37 + 103.7 MΩ). The mean cell diameter for both control and DNBS neurons was similar (control: 19.04 ± 1.13 pF *vs* DNBS: 19.5 ± 0.95 pF). These results indicate that colonic inflammation does not affect the passive membrane properties of sensory neurons in the DNBS-treated group, but it does affect neuronal excitability properties, making them more hyperexcitable.

**Figure 4:**
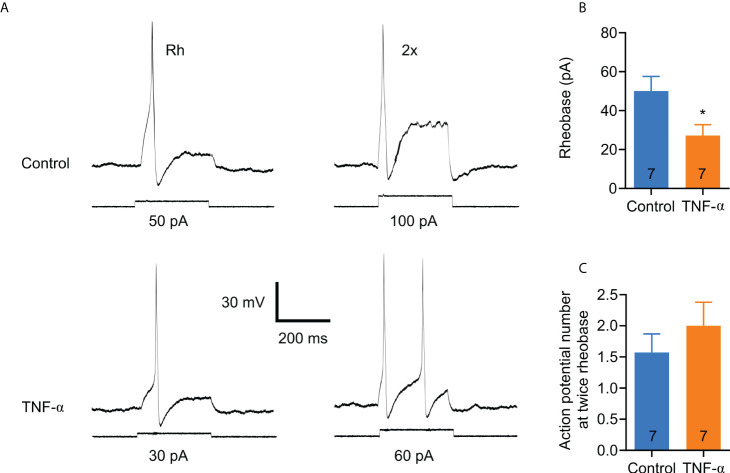
TNF-α increases the excitability of small colonic DRG neurons in a similar manner to those from DNBS-induced colitis mice. **(A)** Representative patch clamp recordings from a control neuron and one incubated overnight with TNF-α (0.1 μg/ml). The traces on the left are action potentials generated by the minimum applied current (rheobase), and the traces on the right are the action potentials generated by twice the rheobase. **(B)** Bar graph of summary data showing that neurons incubated overnight with TNF-α (0.1 μg/ml) exhibit a significant reduction in rheobase compared to neurons incubated with medium alone. The numbers inside the bars represent the number of neurons recorded, and the bars represent the mean ± SEM. The * indicates statistically significant (P < 0.05) for the unpaired t-student test. **(C)** Bar graph of the summary data of the number of action potentials at twice the rheobase. No significant changes are observed. The numbers inside the bars represent the number of neurons recorded, and the bars represent the mean ± SEM.

**Figure 5:**
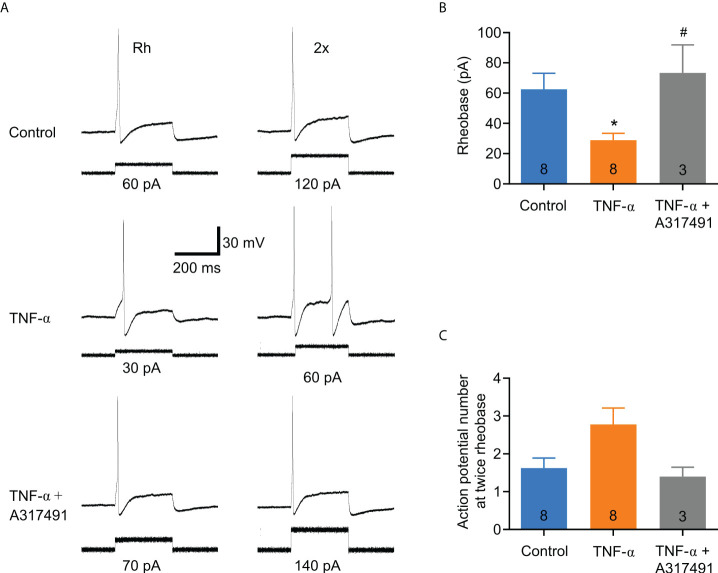
The effect of TNF-α on DRG neuronal excitability is markedly attenuated by the selective P2X3 receptor antagonist, A-317491. DRG neurons were co-incubated with ATP (100 µM), TNF-α (0.1 µg/ml) and the P2X3 receptor antagonist A-317491 (1 µM). The antagonist was incubated for 30 min before applying ATP and TNF-α. **(A)** Representative Patch Clamp recordings from a control neuron (ATP alone), one incubated overnight with ATP plus TNF-α (0.1 μg/ml), and one incubated with ATP plus TNF-α + A-317491 (1 μM). The traces on the left are action potentials generated at the minimum necessary current (rheobase), and the traces on the right are the action potentials generated by twice the rheobase. **(B)** Bar graph of summary data showing that the excitatory effect of TNF-α on sensory neurons is prevented by the P2X3 receptor antagonist, A-317491. The numbers inside the bars represent the number of neurons recorded, and the bars represent the mean ± SEM. The * indicates statistical significance (P < 0.05) by one-way ANOVA with a Bonferroni post hoc test. **(C)** Bar graph of the summary data of the number of action potentials at twice the rheobase. No significant changes are observed. The numbers inside the bars represent the number of neurons recorded, and the bars represent the mean ± SEM. The symbol # denotes a statistically significant difference P ≤ 0.05 when comparing the TNF-α group vs. TNF-α plus A317491group.

In order to determine whether TNF-α is capable of producing similar changes in excitability in DRG neurons from DNBS-treated mice, patch-clamp recordings were executed from dissociated T8-L1 DRG neurons incubated overnight with TNF-α (0.1 μg/ml). Like DRG neurons from DNBS-treated mice, the rheobase was significantly decreased in TNF-α-incubated neurons (representative recordings shown in **Figure 4A**), which was reduced by 54.3% (TNF-α: 27.1 ± 5.7 pA, *n* = 7; *vs* control: 50 ± 7.6 pA, n= 7; *P* = 0.03; unpaired student *t*-test; **Figure 4B**). The number of action potentials elicited at twice rheobase was almost the same between neurons incubated with TNF-α and neurons incubated with a culture medium. To test whether P2X3 receptor activation precedes the potentiation of DRG excitability by TNF-α, neurons were co-incubated with ATP (100 μM), TNF-α (0.1 μg/ml) and the P2X3 receptor antagonist A-317491 (1 μM). The antagonist was applied 30 min prior to ATP and TNF-α. The effects of TNF-α on the rheobase of neurons were inhibited by pre-incubation with A-317491 (**Figure 5A**). The mean rheobase of control neurons (ATP alone) was 61.3 ± 9.8 pA, while TNF-α-incubation decreased this to 28.8 ± 5.1 pA, a response that was blocked by pre-incubation with A-317491 followed by TNF-α stimulation, resulting in rheobase of 73.3 ± 18.5 pA (**Figure 5B**). Between these groups, there were no differences in the number of action potentials elicited with a stimulus twice rheobase (**Figure 5C**). Taken together, these results indicate that TNF-α enhances DRG neuron excitability, potentially through modulation of P2X3 receptors.

### Colitis-associated P2X3 receptor overexpression in T8-L1 DRG neurons

**Figure 6:**
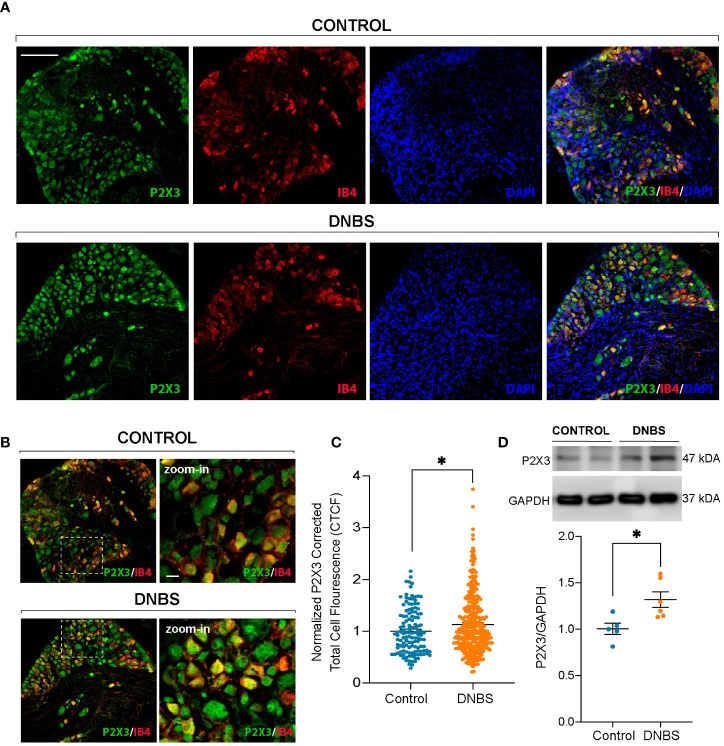
P2X3 receptor (green) and IB4 (red) immunoreactivity in T8-L1 DRG neurons in control mice and after the fourth day of induction of DNBS-induced colitis **(A)**. Note the increase in P2X3 immunoreactivity showing colocalization with IB4+ neurons in the DNBS group compared to the control group **(B)**. Scale bar 100 µm and zoom-in 15 µm. **(C)** P2X3 fluorescence intensity was quantified using ImageJ, and the corrected total cell fluorescence (CTCF) values were converted into fold change values and represented as scatter bars. Data are shown as mean ± S.E.M. * P < 0.05 significant compared with respective control by unpaired t-test. **(D)** Above: Representative immunoblots depicting the protein levels of P2X3 receptors from T8-L1 DRGs from the control group and DNBS group mice. Upper graph: Histogram showing the immunoblot band density of P2X3 receptor protein. The expression of P2X3 receptor protein was significantly increased in DRG neurons from DNBS-treated mice. Data are shown as mean ± S.E.M. * P < 0.05 significant compared with respective control by unpaired t-test.

The electrophysiological data described above suggest that an inflamed colon may cause direct sensitization of primary afferent neurons through direct modulation of P2X3 receptors. This direct modulation could be a result of DRG neuron P2X3 receptor overexpression. To test this hypothesis, we carried out immunofluorescence and western blot assays. It is well known that the majority of P2X3+ DRG neurons in the mouse are non-peptidergic neurons that bind to isolectin B4 (IB4) (28–30). Representative immunofluorescence images are shown in **Figures 6A, B**. Immunofluorescent analysis of P2X3 receptors in T8-L1 DRGs revealed that compared to controls, the P2X3-IR measured as Corrected Total Cell Fluorescence (CTCF) was markedly increased in DNBS-treated mice compared to control mice (*P* < 0.05; **Figures 6A**–**C**). Western blot analysis confirmed this finding, as P2X3 protein levels in T8-L1 DRGs significantly increased in mice with DNBS-induced colitis (*P <*0.05; **Figure 6D**). These results reveal an upregulation of P2X3 receptor in DRG neurons that innervate the colon of DNBS-treated mice.

## Discussion

Sensory neurons are important regulators of diverse pro-inflammatory conditions. For example, sensory neurons within melanoma tumors are thought to be beneficial for the patients, as they have been linked to a reduction in cancerous cell growth (31) *via* activation of the immune system in the tumor niche (32). Gut sensory neurons, in specific, can affect intestinal physiology and be affected by systemic effects. It is well documented that there is a marked decrease in nerve satiety signaling in jejunal sensory neurons of obese animals (33), and recently the glucagon-like peptide 1 receptor (GLP1R) presence in gut afferents has been implicated in the maintenance of glycemia during food intake (34). Colon afferent neurons of the dorsal root ganglia become hyperexcitable when confronted with bacterial cell products (LPS) *in vitro* (35) and *in vivo* (36), a condition that translates to augmented nociception, or increased perception of pain. Interestingly, this effect can be suppressed by healthy, commensal microbiota (37) or by direct targeting of the TRPV1 channel (38). Sensory neurons, therefore, can affect and be affected by local, distal, or even systemic physiological events.

Previous studies demonstrate that there is an increase in TNF-α liberation in acute colitis (23), and we hereby provide evidence that the P2X3 receptor expression increases in response to DNBS-colitis. Thus, the results of this study support the hypothesis that the release of pro-inflammatory mediators, probably TNF-α, during acute colitis contributes to the up-regulation of P2X3 receptors causing hyperexcitability in small-diameter DRG neurons innervating the inflamed colon, which may lead to nociceptive signals in the central nervous system (CNS) and contribute at least in part to nociceptive plasticity of visceral afferents. In support of this interpretation, previous reports have described the involvement of the P2X3 receptors in visceral hypersensitivity during acute TNBS-induced colitis (12, 39).

The inflammatory microenvironment of the DRG nociceptive neuronal axon terminals is composed of numerous inflammatory mediators, many with the capacity for neuronal activation or sensitization (7). The mediator identity, production, and secretion rely on the inflammatory setting characteristics. Given this variability, the first objective of this study was to characterize the inflammation and determine which of the classical acute-phase pro-inflammatory cytokines were expressed. As previously published, DNBS requires 1-3 days in order to produce consistent inflammation; thus, animals are frequently sacrificed 4-days post-DNBS administration (13, 40). Indeed, intestinal inflammation was observed to be in an active phase four days after DNBS application, with a significant increase in TNF-α mRNA expression. Several studies have consistently shown that lymphocytes and antigen-presenting cells (APCs) orchestrate the inflammatory process in active IBD, mainly through TNF-α production, which has been correlated to the endoscopic grade of inflammation (3, 41).

Small DRG neurons from DNBS-treated mice were hyperexcitable, as their rheobases were lower compared to the control counterparts, revealing, thus, an effect of the inflammatory environment on the neuronal function. This alteration in threshold likely renders these neurons more sensitive to sub-threshold stimuli *in vivo* under inflammatory conditions. This finding is consistent with previous studies, revealing that intestinal inflammation induced hyperexcitability in colonic small DRG neurons (42, 43). Furthermore, we found that DNBS-induced hyperexcitability of T8-L1 DRG neurons is similar to that observed in those T8-L1 DRG neurons incubated with TNF-α. These results highlight the important role that TNF-α plays in colitis nociception. Our results agree with another study showing that DRG neurons co-incubated with TNF-α and supernatants from patients with active UC displayed significantly low rheobases and augmented number of action potential discharge compared to control neurons (23). Unlike the aforementioned researcher, there was no difference in number of action potential discharges. This discrepancy could be because we only incubated with TNF-α, and therefore we did not observe the synergistic effect of the additional pro-inflammatory mediators found in the supernatants of UC patients.

The mechanism by which TNF-α has pronociceptive effects on colonic DRG neurons is not fully understood. There is an increasing body of evidence demonstrating that TNF-α exerts its effects directly on primary afferent neurons to induce hypersensitivity (23, 44, 45) through ion channel modulation, this includes the capsaicin receptor TRPV1, and voltage-gated Na^+^, K^+^, and Ca^2+^ channels (43, 46–48). On the other hand, there are also studies showing that homomeric P2X3 and heteromeric P2X2/3 receptors affect the development of inflammatory hyperalgesia (10, 12, 49, 50). To investigate whether TNF-α increases the excitability of DRG neurons through P2X3 receptors modulation, neurons were pretreated with the P2X3 receptors antagonist A-317491 before applying ATP and TNF-α. Our results demonstrate, for the first time, that pre-incubation with A-317491 prevents the increase in neuronal excitability induced by TNF-α. This suggests that TNFα triggers sensory neuron hyperexcitability through direct P2X3 receptor modulation. There is data supporting this notion, as TNF-α has been demonstrated to activate a p38 MAPK-dependent pathway that leads to the quick P2X3 sensitization in DRG neurons (51).

Augmented ATP responses in DRG neurons treated with TNF-α likely to arise from upregulation of P2X3 receptor expression. We have demonstrated that P2X3 upregulation occurs in neurons of the T8-L1 DRGs (these are known sensory neurons of the distal colon and rectum) posterior to colitis-induction (52). This P2X3 over-expression coincides with IB4-positive neurons, suggesting that they are nociceptive neurons of non-peptidergic nature sensing colorectal mechanical nociception (53). Results like P2X3 receptor overexpression has also been found in DRG neurons after TNBS colitis induction (39). These studies indicate a primary role of P2X3 receptors in nociceptor sensitization during acute inflammatory colitis.

Our findings provide new insight into ulcerative colitis and provide evidence that pro-inflammatory mediators such as TNF-α enhance sensory neuron excitability of primary afferent fibers innervating the colon through modulation of P2X3 receptors activity. Moreover, these findings highlight the need to develop selective P2X3 antagonists with the aim of using them as therapy in colonic inflammatory diseases.

## Conclusions

In conclusion, our results suggest that, during acute colitis, the release of pro-inflammatory mediators, such as TNF-α, increases the excitability of DRG sensory neurons whose peripheral nerve endings innervate the colon. This effect is reversed by a selective P2X3 receptor antagonist, suggesting that TNF-α, through modulation of P2X3 receptors, causes increased excitability of sensory neurons in an acute colitis model.

## Data availability statement

The raw data supporting the conclusions of this article will be made available by the authors, without undue reservation.

## Ethics statement

The animal study was reviewed and approved by Ethics Committee to use animals in teaching and research at the Autonomous University of Aguascalientes (CEADI-UAA 5/03/2018).

## Author contributions

EV-M performed the research, formal analysis, investigation, visualization, and supervision. CS-N performed the research and analyzed the data. PB-I and DR-P carried out the immunofluorescence and western blot experiment and analyzed the data. TB-G performed the cell DRG cultures. FO-C, AB-E, and PB-I wrote, revised & edited the paper. RG-A carried out conceptualization of the project, the methodology, validation, formal analysis of results, acquisition of resources, supervision of personnel, project administration, funding acquisition, and writing the original manuscript draft. All authors contributed to the article and approved the submitted version.

## Funding

This work was supported by Project Num. PIFF17-1 from the Autonomous University of Aguascalientes and by Project Num CF19-21854 from CONACYT, Mexico. RG-A was supported by SEP- PRODEP. PB-I was supported by SEP-PRODEP and the International Association for the Study of Pain (IASP) through an early career research grant.

## Conflict of interest

The authors declare that the research was conducted in the absence of any commercial or financial relationships that could be construed as a potential conflict of interest.

## Publisher’s note

All claims expressed in this article are solely those of the authors and do not necessarily represent those of their affiliated organizations, or those of the publisher, the editors and the reviewers. Any product that may be evaluated in this article, or claim that may be made by its manufacturer, is not guaranteed or endorsed by the publisher.
